# Understanding the Behavioural and Social Drivers of Childhood Vaccination Uptake Among Caregivers: A Qualitative Study in Cape Town, South Africa

**DOI:** 10.3390/vaccines14040320

**Published:** 2026-04-03

**Authors:** Lindi Mathebula, Charles S. Wiysonge, Sara Cooper

**Affiliations:** 1 Cochrane South Africa, South African Medical Research Council, Cape Town 7501, South Africa; 2 Division of Epidemiology and Biostatistics, Department of Global Health, Stellenbosch University, Cape Town 7505, South Africa; 3 School of Public Health and Family Medicine, University of Cape Town, Cape Town 7700, South Africa

**Keywords:** childhood vaccination, childhood immunisation, vaccine uptake, vaccination coverage, behavioural and social drivers (BeSD), caregiver determinants

## Abstract

**Background**: Childhood vaccination remains the cornerstone of public health strategies, substantially reducing global morbidity and mortality, yet suboptimal uptake persists in many settings. In South Africa, the challenge is evident, with persistent outbreaks of vaccine-preventable diseases. Addressing localised immunisation shortfalls requires elucidating the complex interplay of factors beyond conventional access barriers. This qualitative study provides context-specific insights into the behavioural and social drivers influencing childhood vaccination uptake among caregivers in Cape Town, South Africa. **Methods**: Utilising an exploratory qualitative research design, thematic analysis was applied to interview data (*n* = 25 caregivers) collected via a purposive sampling strategy designed to capture maximum variation in experiences within targeted low-uptake subdistricts. Interpretation of the data was systematically guided by the World Health Organization’s Behavioural and Social Drivers (BeSD) framework. The latter consists of four domains, namely, “Thinking and Feeling”, “Social Processes”, “Motivation”, and “Practical Factors”. **Findings**: Analysis across BeSD domains reflected a pattern of the intention–behaviour gap, where caregivers are motivated for vaccination but face structural and practical barriers affecting timely uptake. In the Thinking and Feeling domain, widespread conviction regarding the vital benefits of vaccination co-existed with significant anxiety concerning minor side effects (e.g., pain and fever), which sometimes precipitated missed subsequent appointments. Caregivers frequently accept immunisation as a social routine despite having limited knowledge of the diseases it prevents. Social Processes demonstrated that while decision-making authority rested primarily with mothers, compliance relied on the delegation of logistical responsibilities to extended family members. Critically, reports of poor communication, judgment, or negative attitudes among healthcare workers undermined trust and acted as barriers to sustained engagement. Within the Practical Factors domain, structural constraints frequently overshadowed high intent, with pervasive issues such as long waiting times and financial costs cited as the main reasons for missed appointments. **Conclusions**: Participants generally expressed strong acceptance of vaccination, but attainment of optimal coverage is constrained by systemic failures in patient–provider communication and persistent logistical barriers within the public healthcare delivery system. Strategic public health interventions must therefore move beyond addressing only attitudinal opposition to prioritise targeted efforts that mitigate structural constraints and reinforce personalised, empathetic communication to sustain caregiver confidence and adherence.

## 1. Introduction

Vaccination efforts have saved millions of lives and prevented countless illnesses and disabilities worldwide since they were introduced over fifty years ago [1,2]. These efforts were officially organised globally with the introduction of the Expanded Programme on Immunisation (EPI) by the World Health Organization (WHO) in 1974 [3]. Despite these significant achievements, many areas worldwide still fall short of the immunisation coverage goals set by initiatives like the Sustainable Development Goals (SDG3) [4]. Globally, vaccination coverage with the third dose of diphtheria-tetanus-pertussis containing vaccine (DTP3) remained at 84% in 2023 [4]. Meanwhile, the impact of the COVID-19 pandemic led to 67 million children missing their routine immunisation schedules [5].

The difficulty of maintaining high immunisation coverage is especially evident in many low- and middle-income countries (LMICs) such as South Africa, where coverage remains below ideal levels [6,7]. In 2024, South Africa’s national DTP3 coverage was 84%, which is below the 90% target established by the Global Vaccine Action Plan (GVAP) [8]. This inadequacy has precipitated the re-emergence of vaccine-preventable diseases (VPDs), such as recent measles and diphtheria outbreaks observed in various provinces, including the Western Cape Province and the Cape Town Metropolitan Health District [9]. While the Western Cape Province has reported higher vaccination coverage than the national average, some areas within the Cape Town Metropolitan Health District still show pockets of lower uptake [10]. The 2018/19–2020/21 Cape Town Metro Health Plan showed that vaccination coverage for children under 1 year old ranged from 74% to 88% across health subdistricts in 2018 and 2019, which was significantly below the target threshold [11].

Achieving and maintaining high vaccination coverage involves recognising that barriers go beyond just logistical issues, such as insufficient vaccine supply and weak infrastructure [12]. Recent evidence increasingly highlights the decisive influence of psychosocial factors, especially vaccine hesitancy, described as a motivational state of being unsure about or opposed to vaccination despite its accessibility [5,13]. Vaccine hesitancy is characterised by complex determinants, such as complacency, convenience, and confidence, posing a significant threat to vaccination success worldwide, including in African countries [13]. Given that vaccine decision-making is inherently complex and varies by context, accurately identifying localised barriers is essential to creating targeted, effective interventions [14].

To systematically understand these complexities, the WHO established the Behavioural and Social Drivers (BeSD) of vaccination framework. This framework provides standardised, validated tools for measuring the modifiable drivers of vaccine uptake, moving beyond limited measures of vaccine confidence to capture a broader range of determinants [15]. Vaccination behaviours are often influenced by a complex interaction between structural service factors and behavioural and social determinants. The BeSD framework conceptualises these influences across four domains: thinking and feeling, social processes, motivation, and practical issues related to access and service delivery [16].

While previous studies have examined vaccination coverage and determinants of uptake in South Africa, fewer studies have explored the behavioural and social drivers shaping caregivers’ vaccination decisions in urban contexts using qualitative approaches. Understanding these perspectives is essential for designing contextually appropriate interventions to improve vaccine uptake [17]. More specifically, previous research in the Cape Town Metropolitan Health District has either narrowly examined healthcare workers’ perceptions or studied vaccine hesitancy among other adult groups, leaving a significant gap in understanding the views of the main users of childhood immunisation services [13,17,18]. This study (phase 2) is part of a three-phase mixed-methods research using the BeSD framework, as outlined in the published protocol [19]. It aims to fill an important gap in the evidence base and to explore the behavioural and social factors influencing childhood vaccination in the Cape Town Metropolitan Health District. This qualitative study aimed to explore behavioural and social drivers influencing childhood vaccination uptake among caregivers of children aged 0–2 years in selected subdistricts of the Cape Town Metropolitan Health District, using the WHO Behavioural and Social Drivers framework.

## 2. Methods

This study employed a qualitative research approach based on the WHO BeSD conceptual framework for behavioural and social drivers of vaccine uptake. Given the variation across health subdistricts in the Cape Town Metropolitan Health District, this study focused on three subdistricts with low vaccination coverage to explore the behavioural and social drivers of childhood vaccination uptake [11]. The Eastern subdistrict is characterised by high-density informal township areas with low-income housing and limited access to services [20]. Mitchells Plain subdistrict features a mix of densely populated urban, formal suburbs, and low-income neighbourhoods, including large townships with diverse socio-economic conditions [20]. Finally, the Tygerberg Subdistrict is characterised by more developed urban and suburban areas with middle to high-income infrastructure and reliable service access [20]. The study population consisted of caregivers of children aged 0–2 years from the three selected subdistricts of the Cape Town Metropolitan Health District who had participated in Phase 1 of the mixed-methods study, a caregiver survey to estimate the rate of childhood vaccination uptake and assess associated factors [19].

### 2.1. Sampling

The qualitative interviews formed part of an explanatory substudy within a sequential mixed-methods design. Inclusion criteria for participation in the qualitative interviews were caregivers who had previously participated in the Phase 1 survey, had a child aged 0–2 years, and had consented to be contacted for follow-up research. We excluded caregivers who did not meet these criteria, were unavailable at the time of recruitment, or declined to participate in the qualitative interview. A maximum variation purposive sampling approach was employed to select a sample of caregivers. Maximum variation purposive sampling is used when one wishes to ensure that one’s sample comprises stakeholders that potentially differ along various important dimensions [21]. We used this approach to ensure that the sample reflected a range of socio-demographic characteristics, including those related to geographical location, education, socioeconomic status, and gender. Drawing on initial findings from the survey (Phase 1 study), we also sought to ensure that caregivers with a range of views and practices regarding childhood vaccination were included [22]. This included individuals who had accepted vaccination for their children, those who were hesitant, and those who had previously refused vaccination for their children. We continued interviewing participants until data saturation was reached, meaning that no new themes were emerging, leading to a final sample of 25 participants [23]. Saturation was determined analytically rather than numerically, based on thematic redundancy observed during concurrent data collection and analysis.

### 2.2. Recruitment and Enrolment

The principal investigator (PI) and field research assistants contacted selected participants to invite them for an interview using the contact phone number provided during the survey in the phase 1 study. Upon invitation to the study, participants were provided again with the information sheet about the study, which was explained verbally or sent via WhatsApp in one of three languages: English, IsiXhosa, or Afrikaans. The participants were also given the opportunity to contact the principal investigator or field research assistants with any questions. Caregivers were also provided with consent forms, which were explained to them in their preferred language from the three available options. Participants were enrolled in the study once their consent was obtained.

### 2.3. Data Collection

The PI and field research assistants conducted semi-structured interviews using an adapted BeSD Childhood Immunisation In-Depth Interview Guide (CIDI) for Caregivers with caregivers of children aged 0–2 years [15]. The PI piloted the topic guide among two participants to test the flow of the questions, the relevance of probes, and the feasibility and acceptability in terms of time and length of the discussion. The pilot study’s results informed the adaptation of the guide. Each interview was conducted in a private room, at a convenient time and place for the participant. Interviews were conducted by the PI or a field research assistant in the participant’s preferred language. The interviews lasted an average of 20 min, although some interviews extended beyond this depending on participant responses. As the semi-structured interview guide focused on key domains of the BeSD framework, this allowed participants to discuss relevant experiences and perceptions in a structured but flexible manner. Participants were asked questions structured around the four domains of the BeSD—Thinking and Feeling, Social Processes, Motivation, and Practical Issues—to understand their experiences with and perceptions of childhood vaccinations. Interviews were conducted in participants’ preferred language (English, isiXhosa, or Afrikaans). Those in isiXhosa and Afrikaans were audio-recorded, transcribed verbatim, and translated into English by fluent speakers. Back-translation was not performed. To ensure semantic accuracy, the research team reviewed the translated transcripts for clarity and consistency, focusing on preserving the original conceptual meaning during the review translation.

### 2.4. Data Management

All personal identifying information was removed from transcripts. Each interview was assigned an identification number, which was used instead of the participants’ names on hard copies and electronic documents. Study notes from discussions and recording devices were securely stored in a locked cupboard in the principal investigator’s office. The audio recordings were uploaded to a secure, password-protected iCloud location at the end of each interview. Electronic files, such as transcripts, were password-protected and stored in the password-protected computers of the principal investigator and her supervisors. A backup of electronic data was saved on the password-protected iCloud location. All hard copies, including handwritten interview notes, printed transcripts, and signed consent forms that contain identifying participant information, were stored in a locked cabinet accessible only to the principal investigator. The PI securely stored all records after the study concluded and will retain them for the period required by the University. Data from this study has been restricted to the principal investigator, her supervisors and the research assistant.

### 2.5. Data Analysis

The anonymised transcripts were downloaded into NVivo 15, a software programme by QSR International that aids with the management and analysis of qualitative data [24]. The data were then analysed through a thematic analysis approach using the phases described by Braun and Clarke [25]. Thematic analysis is a useful method for identifying and describing recurring patterns in data. A thematic analysis can either provide a rich overall portrayal of the dataset or give an in-depth analysis of either one or a few aspects of the material [26]. The analysis sought to develop themes related to caregivers’ experiences and perspectives of childhood vaccination, their attitudes towards childhood vaccination uptake, and the behavioural and social factors influencing their decisions to vaccinate or not.

Data were analysed using thematic analysis. An initial coding framework was developed based on the BeSD domains; however, coding also remained open to inductive identification of additional themes emerging from the data. The PI began the analysis by reading and re-reading the transcripts, noting down preliminary ideas. After this familiarisation with the data, she then developed an initial coding framework using the four domains of the BeSD framework and the specific questions of the BeSD interview guide as themes and subthemes, respectively. The PI and another team member then used this framework to conduct line-by-line coding of two transcripts, noting potential themes and subthemes that did not fit into the initial framework. They then compared and contrasted their coding to develop a revised coding framework. The PI then coded the rest of the transcripts, which were reviewed by the supervisory team. During this process, additional or revised codes were developed iteratively as determined by the data and added to the coding framework. Generated codes and themes were independently verified against raw data for accuracy and consistency. The PI then developed the themes and codes, along with illustrative quotes, to produce analytical summaries. The remaining transcripts were coded by the PI with a refined framework. Coding and interpretations were regularly reviewed with the supervisory team for consistency and rigour. Analytic notes documented evolving interpretations to ensure transparency in the development of themes.

### 2.6. Reflexivity

This qualitative component used an interpretive approach to understand caregivers’ views on childhood vaccination. Interviews were conducted by the PI and trained research assistants using a semi-structured guide based on the BeSD framework. The PI recruited experienced qualitative research assistants and ensured they received formal training in qualitative methods before beginning the study. The research team was aware that interviewer characteristics and positionality could influence participant responses. To reduce potential bias, interviewers received training on neutral questioning techniques and maintaining a non-judgmental stance during interviews. The use of a standardised interview guide also helped ensure consistency across interviews. The PI aimed to report on methodological, interpersonal, and contextual reflexivity, including transparency of methodological choices, power dynamics with participants, and how context influenced the research findings. The research team kept reflexivity journals with notes and reflections after each interview to consider interview techniques and underlying dynamics.

## 3. Results

### 3.1. Description of Participants

Twenty-five caregivers from the three selected subdistricts of the Cape Town Metro District were interviewed. Most caregivers interviewed are primarily responsible for children under 2 years old, although some also care for older children or have multiple children in their care. The living situations described by the caregivers were diverse, ranging from small family units to large, multigenerational households with some exceeding 10 household members. Table 1 below shows the demographic characteristics of the included caregivers. Many of the caregivers reported that their children were up to date with immunisations, while some had missed vaccinations due to various reasons. The majority of the caregivers were parents of the children, with one being a father. Additionally, two interviewees were family members and not parents.

### 3.2. Social and Behavioural Drivers of Vaccination

The thematic analysis identified several key themes across the four domains of the BeSD framework. An overview of the themes and subthemes is presented in Table 2. Caregivers shared common concerns and experiences related to vaccination and the practical aspects of accessing services. Some also made recommendations regarding how barriers might be addressed. We have organised these findings according to the four domains of the BeSD framework, which we present below.

### 3.3. Thinking and Feeling

For this domain, caregivers’ perceptions mainly centred on their understanding of vaccines and their benefits, general anxieties about their child’s health, and concerns about vaccination side effects. For many caregivers, there was a widespread belief that vaccination is vital to prevent diseases, which in turn supports the child’s overall well-being, often accompanied by a sense of relief and safety when a child is vaccinated. That said, concerns about side effects were ubiquitous amongst caregivers, which for some contributed to hesitancy towards their child getting vaccinated.

#### 3.3.1. Importance and Benefits of Vaccination

The large majority of caregivers interviewed generally perceived childhood vaccination as necessary for their child’s health, as they viewed vaccines as a means of protecting their child against diseases, and they often connected this to the overall well-being of their child. Many caregivers expressed a strong belief in the protective value of vaccination. This perception appeared to reinforce their willingness to vaccinate despite limited knowledge of specific diseases prevented by vaccines. As one caregiver explained:

“Then I feel at ease, because you know, the vaccines let them, they grow and prevent them from getting sick and all those stuff”

(Caregiver 02)

Many of the caregivers shared these sentiments, indicating that they make decisions based on what they believe is right and safe for their child. Some caregivers drew specifically on their personal experiences with illness or a general understanding of the protective role of vaccines. As this caregiver explained:

“I had to think about what is for my son and for his health. But at the end of the day, I just try to tell myself for the good of your child. It’s for the health of your child. I just put the positives in my aid”

(Caregiver 25)

Similarly, another caregiver speaking from a perceived disease risk indicated:

“I decided because my child was getting sick. He was catching germs from other people, then you don’t so they took him for it, he will be vaccinated at the end of the day”

(Caregiver 11)

A few caregivers during the interviews displayed a lack of understanding about vaccines or immunisations. For some, this confusion appeared to centre around the difference between vaccination and immunisation, whilst others indicated explicitly that they do not understand much about vaccination. However, in both cases, this did not appear to reduce their acceptance of vaccines-they supported it despite their misunderstandings or lack of knowledge. For them, vaccination seemed to be a routine response, something that people just do. For example, one of the caregivers, when she was initially asked about how she feels about her child receiving a vaccination, indicated

“It’s not a good thing. I don’t like it”

(Caregiver 06)

After the interviewer provided a neutral clarification of terminology to ensure a shared understanding of “vaccination” as immunisation, the caregiver clarified her response:

“No, then it makes sense. It’s very important. She is due to go to the hospital now for her injections”

(Caregiver 06)

Another caregiver stated explicitly that she does not know what the vaccine was for, but accepted them for her child nonetheless:

“Because I must do it, I never knew what it was for. I just went because my child was certain months, and I must go, and it’s for vitamins and all that”

(Caregiver 17)

#### 3.3.2. Concerns About Side Effects

Various caregivers expressed concerns about vaccination side effects, ranging from minor to significant concerns about side effects. Many of these caregivers were particularly concerned about their child’s reaction after receiving the vaccine, reportedly becoming quite sick afterwards. Some of these caregivers showed reluctance in their child receiving other vaccines, resulting in missed follow-up scheduled vaccinations. For example, one caregiver lamented:

“The last time I was in was in February, because she had to go this month, so I didn’t go…. because, reasoning being she gets so sick… for about a week. And then I must, like Friday I went to the hospital with her and so they told me they injured a vein.”

(Caregiver 18)

With such an experience, the caregiver showed signs of hesitancy towards the vaccines even when they had previously received other vaccines. In a similar way, another caregiver expressed significant concern about her child receiving other vaccinations after she became ill from a vaccine. As she explained:

“Because they, they make her sick, and then I must go to a hospital to find out and they keep me there for a few weeks, or days”

(Caregiver 18)

For other caregivers, their child getting sick after receiving a vaccine made them question the vaccine or how it was administered. For example, this caregiver expressed her fears:

“Sometimes a little scary, I don’t know if they give the child the right stuff, and sometimes they get a little sick also”

Sharing these concerns, another caregiver wondered whether something had been done incorrectly because her child became sick after receiving the vaccine:

“Because what happens afterwards is big for me. The child could get seriously sick, you know. Which I do not understand. Maybe because the nurse stabbed incorrectly, you never know”

(Caregiver 23)

A few caregivers showed worries about the pain their child experiences when receiving injections, while also indicating some of the things they do to alleviate their child’s pain thereafter. For example, one caregiver explained:

“When we get at home, I give her Panado [paracetamol]. So, then she sleeps for a while. And when it withdraws, and then it starts paining. That’s the moment I give her the Panados”

(Caregiver 05)

Another caregiver recounted doing a similar thing to help ease her child’s pain after receiving the vaccine:

“I always put some menthol camphor on the injection spot, and panados for the pain”

(Caregiver 20)

Other caregivers described doing these kinds of activities in preparation for the vaccine, such as administering pain medication, such as paracetamol, before visiting the healthcare facility. These caregivers were of the opinion that paracetamol would reduce the pain and swelling once their child received an injection.

One caregiver who expressed concerns about vaccination injections proposed an alternative way of giving vaccines to reduce discomfort. She proposes:

“My suggest is that they should turn injections into medicines, because why, sometimes, when children receive the vaccination is not easy to get them quiet again. So it may as well be in the form of medicine, or whatever”

(Caregiver 05)

### 3.4. Social Processes

Most caregivers highlighted the influence of family, friends, and community members on vaccination decisions and practices. While some caregivers revealed openly discussing vaccination among their social circles, others reported a shortage of guidance or awareness about vaccination practices within their communities. Responsibility for childcare within the household, and associated decision-making dynamics, including around vaccination, also appeared to influence caregivers’ vaccination decisions and practices.

#### 3.4.1. Community Norms and Practices

Various caregivers mentioned that they interact with family members and friends about vaccinations. Some described being able to discuss their fears about vaccinating their child with peers, where they receive advice to address these fears. Some caregivers who supported vaccination also mentioned that they often advise their friends and families about the importance of vaccinating their children. For example, one caregiver stated:

“My friends yes, then I will say when it comes to children, we must put their best interest at heart and because I’m older, I will help my friends take their children to take them to the clinic because the health of the child comes first”

(Caregiver 17)

Similarly, another caregiver, when asked what she discusses about vaccines with her friends, described trying to support and teach peers who may be against or hesitant towards vaccines:

“Her response was you dumb… really now they will not do that, they wouldn’t want to aim to kill children or make them sick…… So I try to explain that. To people of female especially, and teach them that and to explain to them to look at the positive side of it and not at the negative”

(Caregiver 16)

However, and in direct contract, other caregivers described feeling isolated when it comes to communicating with friends and family about vaccines, suggesting that they do not receive any advice or guidance in this regard. As one caregiver said:

“No, because people don’t wanna give me advice”

(Caregiver 18)

Some caregivers indicated that they know of groups of parents who do not vaccinate their children because of peer influence. For example, when one caregiver was asked if she was aware whether there are parents who do not vaccinate their children, she answered

“So people don’t, some of them doesn’t want to take the children for the, those vaccination”

(Caregiver 02)

Caregivers also noted that some of the reasons other parents are not vaccinating is due to them being neglectful, lazy to go the healthcare facility, or “because the majority are druggies” according to one caregiver (Caregiver 24)

#### 3.4.2. Delegation of Childcare

Caregivers revealed complex family dynamics around who is responsible for their child’s vaccination. In some cases, other family members besides the mother were reportedly responsible for the child, including ensuring the child is up to date with vaccinations. In other cases, caring for the child, including taking them for vaccination and ensuring they are up to date with vaccination, was a collective responsibility within the household. For example, various caregivers explained that their siblings, uncle or father take the child to the healthcare facility if they are not around. For some caregivers, they emphasise that it is their responsibility to bring their children for vaccination; however, they occasionally delegate this responsibility to family members. As one caregiver explains:

“I normally take my child for the vaccines, and my uncle. Sometimes my uncle and my sister.”

(Caregiver 04)

And another caregiver, when asked the same question, mentions:

“I take him, or if I am at work, I will ask my aunty.”

(Caregiver 22)

One caregiver, who is not the child’s mother, explained that she, along with her other sister, who is also not the child’s mother, ensures the child is up to date with vaccinations. She explains:

“Because her mother is not there. Her mother does not stay with us. Her child lives with me. My sisters help me”

(Caregiver 19)

### 3.5. Practical Issues

For this domain, caregivers described many experiences with the practical aspects of vaccination, which included challenges and satisfactions related to accessibility of the healthcare service, waiting times, healthcare provider conduct, and the overall logistics of attending appointments. While many caregivers expressed appreciation for the efficient services and convenient scheduling provided by the appointment system and reminders for some, the long waiting times and poor communication from healthcare providers appeared to be significant sources of dissatisfaction with the services. Other factors, such as financial barriers and a lack of consistent reminders for others, also appeared to impact on timely attendance.

#### 3.5.1. Accessibility and Travel to the Healthcare Facility

For many of the caregivers interviewed, proximity to the healthcare facility and the availability of transportation were key considerations for attending their vaccination appointments. Most caregivers explained that they walk to the healthcare facility, as most were within walking distance, indicating experiencing an ease of access to the vaccination services. However, other caregivers explained that the health facility is far from their home, and thus they have to take some sort of transport, including taxis or a ride from a family member, which for many was an inconvenience. Some of these caregivers also spoke about the financial constraints this poses and how this is therefore one of the reasons they may skip vaccination appointments and have delays in fully vaccinating their child. This was also linked to the difficulty other caregivers are experiencing accessing healthcare facilities due to the cost of transport. As one caregiver explained,

“I don’t have travelling money. I maybe don’t have food that morning for me to eat, you understand. Then I really just don’t wanna go, because I also don’t have lies for the nurses… you know”

(Caregiver 07)

Several caregivers suggested that proactive outreach from health services is necessary to address logistical and socioeconomic barriers that vulnerable community members often face. They recommended that things such as outreach, mobile clinics and house calls could help ensure that all children receive vaccinations. As one caregiver gave her thoughts:

“Okay, for the children that need to be taken to the clinic I suggest that there be a care nurse that go house to house to check up on those children that need vaccination.”

Another caregiver shared similar thoughts:

“I would actually like it if they open something like that here by us, you know... Here close to us….. Like a mobile thingy….. that would be cool.”

(Caregiver 17)

One of the caregivers expressed concerns over suffering children whose mothers are on drugs and suggested that healthcare providers do home visits to observe what is happening to these children and give vaccinations at home. As one caregiver explains the need for an outreach programme:

“Some parents can’t take to the clinic the mom is sick and the dad as well the children have sores, and they also live will in dirty houses. And these children are sick, and they are infectious.”

(Caregiver 12)

#### 3.5.2. Reminders and Appointment Scheduling

Caregivers listed various methods for remembering appointments or vaccination dates, such as regularly checking the Road to Health Booklet for upcoming dates. A Road to Health Booklet is a booklet used to record the child’s health and development during healthcare visits in South Africa [27]. Some also mentioned receiving SMS reminders or calls to alert them of upcoming appointments. However, caregivers noted that the appointment system is generally ineffective because wait times are very long despite appointments. As one caregiver mentions:

“Sure. Even like they, they give you an appointment for about let me say you might make an example for nine o’clock, then you get help about 11 o’clock. Past 11 to 12.”

(Caregiver 08)

Another caregiver expressed the same sentiment, stating that the appointment system is ineffective.

“I don’t know they have this appointment thing happening, but it’s pointless”

(Caregiver 13)

And similarly, another caregiver mentions:

“Yhor, you just wait extremely long. Even if you have an appointment”

(Caregiver 21)

#### 3.5.3. Long Clinic Waiting Times

Long waiting times at clinics to receive vaccination substantially influenced various aspects of caregiver satisfaction. Many caregivers expressed dissatisfaction with extended wait periods and frustrations with the appointment system, which they felt was only exacerbating the wait times. As one caregiver lamented:

“By the clinic? It’s the long waiting. They tell you this time, and then you arrive that time at the clinic, but then you have to wait very long. The hours are longer of the clinic, that’s what I don’t like”

(Caregiver 04)

Another caregiver, when asked about what they do not like about taking their child for vaccination, explains her frustrations:

“I’m waiting, then I must stand up because the long waiting is irritating the child, from sitting”

(Caregiver 01)

While another caregiver explains the unfairness, including letting other people go before her, while she has been waiting for a long time. She explains:

“Sometimes how long you have to sit there. Sometimes you say “I was first, but why is she going before me”

(Caregiver 21)

#### 3.5.4. Healthcare Provider Communication and Treatment

A few caregivers expressed appreciation for the treatment they received from healthcare providers, including communications about vaccination. As one caregiver indicated,

“They would give the vaccine and any other questions that I have, if I have concerns or so, then I would ask normally the sister and then they would advise me what to do or even say like my son is. In the teething phase that would normally ask him, what do I use for?”

(Caregiver 25)

However, the majority of caregivers spoke about the poor treatment they feel they receive from healthcare providers and the lack of communication they receive about vaccination. For example, one caregiver recounted her recent visit to the clinic:

“Yoooo, but there is a lot of things to say about that clinic. They don’t even if the children had something to eat. I mean, after a certain time children get restless, but they don’t care, they wanna nag and tell you that you need to keep your child”

(Caregiver 02)

In a similar way, another caregiver described how:

“They get some nurses who are very short with you and don’t wanna talk to you, and will not explain something to you”

(Caregiver 05)

Like this caregiver, many others also spoke about the lack of information they receive from healthcare providers. When asked what they dislike about the vaccination service, so many caregivers indicated that healthcare providers do not provide them with sufficient information, which they described as very frustrating. For example, one caregiver emphasised:

“Like I said, they don’t communicate while they’re doing the vaccination... They’re supposed to communicate with the parent, telling them what and what not to do”

(Caregiver 09)

Many other caregivers indicated feeling excluded from the discussion regarding vaccination procedures. Many expressed a desire for better communication and education from healthcare providers about vaccines. In addition to explaining the vaccination process and the risks of vaccination, various caregivers also asked healthcare providers to advise them on managing post-vaccination side effects. According to them, this would help ease their concerns about the vaccination process. Some caregivers suggested the need to improve education beyond traditional written method materials, to more practical, hands-on teaching:

“I would say to educate them and not giving them leaflets or pamphlets because a lot of people don’t read it, but if they can be representative in the clinic, just to give some examples, maybe have a doll and show how vaccinations work, stuff like”

(Caregiver 23)

### 3.6. Motivation

Motivations for caregivers to get their child vaccinated were often associated with the “Thinking and Feeling”, such as personal conviction about the importance of their child’s health and a desire to do what is “right” and “safe”. and “Social Process”, such as their experiences with previous vaccinations, and sometimes “Practical Factors”, including long wait times, which could potentially influence caregivers not to attend vaccination appointments.

#### 3.6.1. Caregiver Motivational States

The majority of caregivers expressed a greater desire to have their child vaccinated. Caregivers often viewed vaccinating their child as highly important, demonstrating their acceptance of the vaccines administered to their child. One caregiver exclaims:

“It is the most important thing to do for a child. Actually, vaccinate your child at the end of the day”

(Caregiver 11)

Some caregivers who are also accepting of the vaccines go on to explain how the vaccines protect the health of their child:

“good because it helps protect them from germs and such”

(Caregiver 12)

Often, caregivers had a positive attitude towards vaccinations, which showed their willingness and intentions to get their child vaccinated. As one caregiver also exclaimed:

“must do it for our kids as it is a good thing for them and keep them healthy”

(Caregiver 03)

Overall, with their intention and willingness, caregivers viewed the vaccination of their child as a necessary and fundamental responsibility, as many expressed how it is their decision and responsibility to ensure that vaccination appointments are honoured. As one caregiver indicated,

“No one did, I registered at my own accord”

(Caregiver 17)

And another caregiver said:

“No, I did it out of my own. I received a date, and then I went”

(Caregiver 07)

#### 3.6.2. Manifestations of Hesitancy

While most caregivers showed high levels of readiness and willingness, some showed a level of hesitancy or a conflicting attitude towards vaccination. Some caregivers mentioned laziness or inconvenience as potential reasons for the delays in vaccination. One caregiver admitted to laziness as a reason for missing vaccinations for her child:

“No, it’s because of laziness [laughter] I am honest”

(Caregiver 22)

Other caregivers mentioned that inconveniences, such as long wait times at the clinic and the need for someone to care for their other children during vaccination appointments, make it difficult for them to attend some appointments. One caregiver explained,”

“So he sits all day, maybe all the time, and then if I go for him, then we go. So that actually takes my time. I also have to go back to my children to see them. There’s no one to look after them for the crisis”

(Caregiver 11)

In contrast, others missed vaccinations due to concerns stemming from previous experiences, such as post-vaccination side effects. As described under the “Thinking and Feeling” domain, such concerns led one caregiver to skip the following vaccination appointment, although it was not an outright refusal to vaccinate her child.

Caregivers also expressed emotional distress and interpersonal conflicts when getting their child vaccinated, such as feeling “hurt” or “sad” when their child was being vaccinated, while others also mentioned their distress with post-vaccination side effects, although this did not deter them from vaccinating their child.

#### 3.6.3. Factors That Sustain Motivation

While most caregivers already expressed high motivation to get their child vaccinated, the interviews also highlight key elements that are mostly mentioned in the “Practical Factors” domain that can reinforce positive motivation for vaccination. Efficient services, for instance, at vaccination centres or primary health facilities, appear to be valued by one caregiver even though they appeared to relate only to specific cases. The caregiver exclaimed:

“What I like about them is they help you very, very quickly, like if I come now and I say he is sick or I check the card, then they will immediately call out his name”

(Caregiver 06)

Quality staff interactions with caregivers also emerged as a positive motivator, as expressed by some caregivers. As one caregiver mentions:

“They give you good service at the clinic, and they treat the baby well”

(Caregiver 10)

In contrast to this approach, several caregivers under the “Practical Factors” domain expressed a dislike for interactions with healthcare providers who do not communicate effectively with them about or during vaccinations. Providing educational information about vaccines and the vaccination process and side effects thereafter appears to foster acceptance of vaccination services. One caregiver explains how she feels about vaccinating her child:

“At this moment, because I understand vaccines better than I did before…. I have, I have a little place and trust in what the sisters are doing and what has been given, and I normally ask the questions what is exactly in the vaccine, so they don’t explain in detail, but I make sure that I on my time do research on it so. It’s just to ease my own mind”

(Caregiver 25)

Overall, the findings illustrate how caregiver motivation to vaccinate children was shaped by a combination of beliefs about vaccine benefits, social influences, and practical experiences with healthcare services.

## 4. Discussion

This qualitative study, guided by the World Health Organization’s Behavioural and Social Drivers framework, sought to explore the complex, context-specific factors influencing childhood vaccination uptake from caregivers’ perspectives in the Cape Town Metropolitan Health District. The use of this comprehensive framework allowed for a detailed understanding of caregiver input across all psychological, social, and logistical factors that influence immunisation behaviours. The findings highlight common barriers across all BeSD areas that subtly challenge the idea of high acceptance levels, despite their widespread presence.

### 4.1. Motivation and the Acceptance Paradox

Caregivers in this cohort demonstrated a reportedly high baseline level of intrinsic motivation and intent to vaccinate. The findings suggest that many participants in this study perceived vaccination as a socially expected behaviour within their communities, as was found in our previous phase 1 study, where a substantial majority of caregivers expressed that they were “very willing” to ensure their child was fully vaccinated. The interviews in this current study revealed that caregivers viewed vaccination as a necessary and fundamental parental responsibility undertaken of their “own accord”. This widespread, positive disposition aligns with previous findings indicating high acceptance rates for routine childhood vaccines in South Africa [4,28]. However, this robust intent does not guarantee sustained adherence to the comprehensive immunisation schedule, indicating the possibility of the well-documented intention-behaviour gap characteristic of public health challenges [6,14]. While explicit refusal was rare, adherence suffers from motivational lapses, sometimes manifesting as acknowledged “laziness” or inconvenience. Furthermore, some caregivers’ concerns about past side effects were sufficient to cause them to miss the next vaccination dose for their child. This shows that even with good intentions for vaccination, fears about vaccine safety remain a major obstacle to complete vaccination.

### 4.2. Thinking and Feeling

The domain of Thinking and Feeling revealed a duality: high conviction in the perceived benefits of vaccination juxtaposed with anxiety regarding perceived harms. Caregivers expressed a widespread belief that vaccination is vital to prevent diseases and safeguard their child’s “overall well-being” [4,28]. This strong positive belief serves as a primary driver for acceptance. Conversely, acceptance is influenced by widespread emotional concerns, mainly focusing on the fear of common minor side effects, such as pain and fever [6]. This distress over perceived post-vaccination sickness sometimes led to reluctance or admission of skipping subsequent appointments [29]. This outcome highlights the need for targeted health communication focused on addressing these particular, localised anxieties, ensuring that caregivers are advised on managing expected reactions and maintaining confidence. Furthermore, despite their willingness, many caregivers reported significant gaps in their knowledge of specific vaccines or the formal immunisation schedule, accepting vaccination instead as a mere normative routine.

### 4.3. Social Processes

Social factors critically mediate immunisation decisions. The previous Phase 1 of the study depicted that the burden of responsibility and decision-making authority rested predominantly with mothers in this cohort [22]. However, practical needs often meant that the limited, yet crucial, roles of extended family were necessary, with caregivers saying they delegated child escort duties to siblings, aunts, or others when mothers could not be present because of work or other constraints.

The immediate social network of peers emerged as a source of positive reinforcement, with caregivers engaging in discussions to mitigate fears and actively advising hesitant friends and family, thereby reinforcing a pro-vaccination social norm. Yet, a significant barrier arose from interactions with frontline healthcare workers (HCWs). While HCWs are globally regarded as the most trusted source of vaccine information, caregivers frequently reported experiences of “poor communication” and feeling marginalised from the discussion regarding vaccine procedures and risks [4,30]. This perceived insufficient clarity and information can erode trust and discourage ongoing engagement, even for highly motivated parents [4].

### 4.4. Practical Factors

The tension between high motivation and inconsistent vaccination may be explained by the domain of Practical Factors, where logistical challenges act as “constraints” that may override favourable intentions [6,14]. The primary sources of dissatisfaction articulated by caregivers were structural and systemic, specifically chronic long waiting times at clinics and ineffective appointment systems, resulting in pervasive time constraints and inconvenience. This reality aligns with other African contexts, where lengthy waiting times and non-integrated services frequently inhibit sustained uptake [4,31]. Furthermore, although geographical proximity afforded many the option to walk to the facility, for those relying on transportation, the added expense transformed the logistical challenge into a critical financial barrier, compelling some to “skip vaccination appointments” [31,32]. Caregivers actively proposed solutions to dismantle these structural barriers, recommending community-based interventions such as expanding “outreach,” “mobile clinics,” or home visits to mitigate financial and access impediments for the most vulnerable populations. They further requested enhanced practical, hands-on teaching from providers, moving beyond mere leaflets to address their information deficits effectively.

### 4.5. Findings in the Context of LMICs and South Africa

The findings of this study align with evidence from other African and LMIC contexts showing that caregivers often express strong motivation toward childhood vaccination while simultaneously facing practical challenges in accessing services [3,31]. Studies conducted in Kenya, Nigeria, and Ghana have similarly reported that logistical barriers, long waiting times, and service delivery constraints can influence vaccination experiences even when caregiver confidence in vaccines remains high [3,31]. Some findings may reflect context-specific features of the Cape Town Metropolitan Health District. As a large urban setting with diverse socio-economic conditions, Cape Town includes both well-resourced urban areas and peri-urban communities facing structural challenges such as transport costs, clinic congestion, and service delivery pressures [20]. These dynamics may shape caregivers’ experiences of vaccination services in ways that differ from rural contexts. Furthermore, structural factors within health systems, including clinic workload, staffing constraints, and communication practices between healthcare providers and caregivers, have been identified in other LMIC settings as important determinants of vaccination experiences [17,18].

### 4.6. Limitations

This study is subject to several inherent methodological limitations common to focused qualitative designs, particularly concerning generalizability and potential for bias. The study population was specifically restricted to caregivers of children aged 0–2 years. This age constraint limits the generalizability of the insights to older children. Moreover, the sample focused predominantly on female caregivers. While this may accurately reflect the gendered nature of healthcare experiences in this context, it necessarily limits the generalizability of the findings and their implications to male caregivers. Because participants were recruited from caregivers who had previously participated in the Phase 1 survey and provided contact information, the sample may overrepresent individuals who are more engaged with health services. The purposive sampling methodology utilised to recruit caregivers from early childhood development (ECD) centres introduced a potential for selection bias. This approach may disproportionately favour individuals who are already motivated to vaccinate their children or actively engage with routine health services, thus limiting the representation of perspectives from those who outright refuse vaccination or are entirely disconnected from the mainstream healthcare system. As is typical in cross-sectional research employing qualitative interviews, the data collected is subject to potential recall bias and social desirability bias. That is, participants may have offered responses that align with perceived social or programmatic norms, rather than revealing their actual behaviours or beliefs. Additionally, the interviews were relatively concise, averaging around 20 min, which might have restricted in-depth discussion on certain topics. Nevertheless, employing a targeted semi-structured interview guide enabled a systematic exploration of key themes concerning the behavioural and social factors influencing vaccination across all participants.

## 5. Conclusions

This qualitative study of caregivers in the Cape Town Metropolitan Health District, informed by the BeSD framework, provides robust, context-specific evidence that distinguishes between high vaccine demand and persistent barriers to uptake. The study finds that, despite high caregiver motivation and acceptance, structural and service-related challenges in healthcare may affect vaccination experiences. This study provides context-specific evidence from an urban LMIC setting, highlighting that barriers to vaccination are primarily structural rather than attitudinal. To improve childhood vaccination rates in Cape Town: leverage caregivers’ motivation by addressing their concerns with culturally relevant dialogue and visuals; reduce administrative barriers, like long waits, and explore flexible delivery methods such as reminder systems and community outreach; and use local evidence to shift from broad policies to targeted, cost-effective actions that tackle specific barriers faced by caregivers.

## Figures and Tables

**Table 1:** Characteristics of Caregivers Interviewed (*n* = 25).

Characteristic	Category	*n* (%)
Subdistrict	Eastern	10 (40)
	Mitchells Plain	9 (36)
	Tygerberg	6 (24)
Relationship to Child	Mother	22 (88)
	Father	1 (4)
	Other family members	2 (8)
Caregiver Age (Years)	18–29	18 (72)
	30–39	5 (20)
	≥40	2 (8)
Education Level	Primary	0 (0)
	Secondary	24 (96)
	Tertiary	1 (4)
Employment Status	Employed	8 (32)
	Unemployed	17 (68)
Child Vaccination Status	Fully vaccinated	14 (56)
	Delayed/missed doses	11 (44)

**Table 2:** Overview of Themes and Subthemes Identified from Caregiver Interviews.

BeSD Domain	Main Theme	Subthemes	Description of Participant Perspectives
Thinking and Feeling	Perceived benefits of vaccination	Protection against illness; Preventing serious disease	Caregivers frequently expressed a strong belief that vaccines protect children from illness and contribute to overall child health.
	Concerns about side effects	Fear of fever or discomfort after vaccination	Some caregivers reported concerns about temporary side effects, such as fever or pain, following vaccination, although these concerns rarely led to refusal.
Social Processes	Trust in healthcare providers	Confidence in nurses; reliance on provider advice	Healthcare providers were often described as trusted sources of vaccination information and guidance.
	Social expectations around vaccination	Community norms supporting vaccination	Many participants described vaccination as a socially expected practice within their communities and families.
Motivation	Parental responsibility	Protecting the child; fulfilling parental duty	Caregivers often framed vaccination as a parental responsibility and an essential part of caring for their child.
	Vaccination as routine practice	Following clinic schedules	Vaccination was frequently described as a routine healthcare activity integrated into early childhood care.
Practical Issues	Health service barriers	Long waiting times; clinic congestion	Participants reported challenges such as long waiting times and crowded clinics when attending vaccination services.
	Access and logistical challenges	Transport costs; scheduling difficulties	Some caregivers described logistical constraints, including transport costs and difficulty attending clinics during working hours.

## Data Availability

All data were collected, stored, and analysed in accordance with Stellenbosch University’s data management policies and the South African Protection of Personal Information Act (POPIA; Act No. 4 of 2013). To ensure confidentiality and anonymity, all personal identifiers were removed from the dataset before analysis, and access was limited exclusively to authorised research team members. Anonymised data will be made available upon reasonable request.
